# Osteogenic Differentiation of Human Mesenchymal Stem Cells in Mineralized Alginate Matrices

**DOI:** 10.1371/journal.pone.0120374

**Published:** 2015-03-13

**Authors:** Marita Westhrin, Minli Xie, Magnus Ø. Olderøy, Pawel Sikorski, Berit L. Strand, Therese Standal

**Affiliations:** 1 Kristian Gerhard Jebsen Center for Myeloma Research, Department of Cancer Research and Molecular Medicine, Norwegian University of Science and Technology, Trondheim, Norway; 2 Centre of Molecular Inflammation Research, Department of Cancer Research and Molecular Medicine, Norwegian University of Science and Technology, Trondheim, Norway; 3 Department of Physics, Norwegian University of Science and Technology, Trondheim, Norway; 4 Department of Biotechnology, Norwegian University of Science and Technology, Trondheim, Norway; Second University of Naples, ITALY

## Abstract

Mineralized biomaterials are promising for use in bone tissue engineering. Culturing osteogenic cells in such materials will potentially generate biological bone grafts that may even further augment bone healing. Here, we studied osteogenic differentiation of human mesenchymal stem cells (MSC) in an alginate hydrogel system where the cells were co-immobilized with alkaline phosphatase (ALP) for gradual mineralization of the microenvironment. MSC were embedded in unmodified alginate beads and alginate beads mineralized with ALP to generate a polymer/hydroxyapatite scaffold mimicking the composition of bone. The initial scaffold mineralization induced further mineralization of the beads with nanosized particles, and scanning electron micrographs demonstrated presence of collagen in the mineralized and unmineralized alginate beads cultured in osteogenic medium. Cells in both types of beads sustained high viability and metabolic activity for the duration of the study (21 days) as evaluated by live/dead staining and alamar blue assay. MSC in beads induced to differentiate in osteogenic direction expressed higher mRNA levels of osteoblast-specific genes (*RUNX2*, *COL1AI*, *SP7*, *BGLAP*) than MSC in traditional cell cultures. Furthermore, cells differentiated in beads expressed both sclerostin (*SOST*) and dental matrix protein-1 (*DMP1*), markers for late osteoblasts/osteocytes. In conclusion, Both ALP-modified and unmodified alginate beads provide an environment that enhance osteogenic differentiation compared with traditional 2D culture. Also, the ALP-modified alginate beads showed profound mineralization and thus have the potential to serve as a bone substitute in tissue engineering.

## Introduction

Bone tissue engineering is the development of materials that can be used to regenerate bone *in vivo*. Bone regeneration strategies can include the use of scaffolds, growth factors, cells or a combination of the three [1]. Still, the golden standard and the most successful treatment of bone defects are autologous bone grafts. The bone graft has osteoinductive properties through the presence of growth factors, vascularization, and by containing cells with osteogenic potential [2]. However, bone grafts are limited in supply and associated with risk of donor site morbidity and infections [3]. Hence, the use of cells with osteogenic potential in combination with scaffolds mimicking bone is a promising alternative to bone grafts.

Mesenchymal stem cells (MSC) are cells that can differentiate into osteoblasts, adipocytes and chondrocytes [4]. Osteoblasts produce bone matrix proteins and they also catalyze the mineralization of bone matrix into bone. Fully mature osteoblasts have a limited life span, and can *in vivo* either be terminally differentiated into osteocytes, that acts as mechanosensors in bone, to inactive bone lining cells or they will undergo apoptosis [5]. A key step to allow survival and osteogenic differentiation of MSC in scaffolds is finding a suitable material that is bioactive, non-immunogenic and that has mechanical properties similar to that of bone [6]. Materials based on extracellular components, such as collagen, are popular choices. However, because these materials lack adequate mechanical properties and because they may stimulate immunogenic responses after transplantation they are not ideal candidates [7]. Miscellaneous inorganic materials such as metals (e.g. titanium) are currently used to fill bone defects and some are used in the clinic [8]. Although such materials may prove sufficiently strong they are poorly incorporated into existing bone and are difficult to combine with cell based tissue engineering approaches. To overcome these problems recent focus has been on making composites by combining both organic and inorganic components [9]. Important factors are the ability to control mineral deposition and distribution to ensure that the composites to largest extent mimic the properties of natural bone or stimulate bone formation. Although an array of polymer/calcium phosphates composites have been developed, many lack the strength, and moreover the toughness and reliability needed for bone tissue engineering [10].

Alginate is a popular candidate polymer in tissue engineering strategies. Alginate is a biopolymer made up by two uronic acids, mannuronate (M) and guluronate (G). Adjacent G-monomers (G-blocks) have high affinity to divalent cations, such as Ca^2+^. The divalent cations crosslink G-blocks in neighboring polymers consequently forming a gel at close to physiological conditions [11]. Alginate is biocompatible, non-immunogenic, thermostable and the composition can be tailored through enzymatic modifications [12–14]. Alginate gels are in general porous hydrogels that allow the transport of oxygen, nutrients and waste to and from encapsulated cells [15]. Alginate gels can be made that protect transplanted cells, i.e. pancreatic islets, against host immune cells and antibodies that allow for transplantation of allogenic and xenogenic tissue without the need for immune protection [11, 16]. Alginate may not meet the mechanical requirements of a bone substitute material, but the polymer's interaction with Ca2+ forms an interesting basis for achieving better control of mineral formation in terms of crystal growth rates, -size and -alignment.

Recently, we have shown that alginate can be mineralized with calcium phosphate, leading to a homogenous and to some extent controllable deposition of mineral phase within the hydrogel network [17–19]. Mineralization is accomplished either by a counter diffusion method [17] or by an enzymatic method where the enzyme alkaline phosphatase (ALP) is utilized to liberate phosphate ions from organic phosphate compounds [19]. Both methods result in nanocrystalline hydroxyapatite (HA) closely integrated in the alginate gel network. The latter method is superior for cell immobilization purposes as lower concentrations of CaCl_2_ are necessary and mineralization can occur over time, as phosphate ions are gradually made available by the encapsulated ALP. In addition, enzymatic mineralization of alginate beads leads to a homogenous distribution of mineral, as opposed to the counter diffusion method where a core-shell distribution of mineral was most prominent [19]. The homogenously distributed mineral was shown to provide a stiffer gel compared to core-shell mineralization, even with lower total mineral content [18]. However, even though the Young’s modulus is higher than in most alginate hydrogel systems [18] the modulus is still low compared with that of natural bone. Therefore these materials are not envisaged for use in load bearing applications as such, but would need to be combined with load bearing cell free scaffold or relay on bone development for mechanical strength.

The fundamentally different microenvironment of a mineralized hydrogel compared to macroporous biomaterials is an interesting feature that may be taken advantage of. Here, we investigated if bone marrow-derived MSCs could survive and differentiate in osteogenic direction in mineralized and unmineralized alginate beads *in vitro*. We also aimed to compare the osteogenic potential of cells embedded in the mineralized and unmineralized 3D hydrogels with cells in traditional 2D culture. Further, changes in the mineral content of the beads during the culture period were studied.

## Materials and Methods

### Alginate solution

Ultrapure high-G alginate (UP MVG, batch: FP-505–01, M_w_ = 238.000 g/mol, [ŋ] = 1105 ml/g, F_G_ = 0.67, F_GG_ = 0.56, N_G≥1_ = 13) purchased from Nova Matrix, Oslo, Norway was used to prepare alginate beads. A 2.0% alginate solution was prepared of alginate in 0.9% (w/V) NaCl, pH was adjusted to 7.2–7.4 and the solution was sterile filtered before further use.

### Cell culture

Human Mesenchymal stem cells (MSCs) obtained from two different male donors (Lonza Inc, Walkersville, MD, USA) were utilized in this study (S1 Table). The MSCs were cultured at 37°C in a humidified atmosphere containing 5% CO_2_ in mesenchymal stem cell growth medium (Lonza). To induce osteogenic differentiation the media was added bone morphogenetic protein 2 (BMP-2, 300ng/mL, R&D Systems, Minneapolis, MN, USA), ascorbic acid (0.05mM), dexamethasone (10^-8^ M) and glycerophosphate (10mM) (hereafter called osteogenic medium). Cells for differentiation purposes were used before passage 8.

### Bead preparation and cell culturing

Encapsulation of cells was performed as described previously [20]. Before encapsulation of cells, Alkaline Phosphatase (ALP, EC3.1.3.1, calf intestinal mucosa, 15 units/mg, Sigma Aldrich, UK) was sterile filtered and mixed with the alginate solution inside a syringe to a concentration of 0.25mg/ml. MSCs was added to the suspension to make a final concentration of 2 x10^6^ cells/mL in a 1.8% alginate solution. The cell/alginate/enzyme solution was subsequently dripped using electrostatic bead generator into a gelling solution containing CaCl_2_ (50mM), NaCl (0.9% w/v) and 4-(2-hydroxyethyl)-1-piperazineethanesulfonic acid (HEPES) (10mM, pH = 7.3). After cell encapsulation, beads were left in the gelling solution for approximately 10 minutes, prior to thorough washings in Hanks Buffered Salt Solution (HBSS, Sigma-Aldrich, St Louis, MO). For mineralization purposes cells received MSC growth medium (Lonza Inc) with added CaCl_2_ (10mM) and β-glycerophosphate (15mM, Sigma) the first 48 hours after encapsulation (henceforth referred to as mineralization medium)). After 48 hrs half of the mineralized beads were given growth medium, while the other half was given osteogenic medium. Both the growth medium and osteogenic medium were added 7.5mM CaCl_2_ for stabilization purposes, as mineralization results in depletion of Ca-ions from alginate gel, and as a consequence slow gel dissolution. Cells were also encapsulated in alginate beads without the use of ALP, and these cells were also cultured in either growth medium or osteogenic medium. Additionally, cells were grown in culture flasks as a comparison to 3D culture. All cultures were maintained at 5% CO2 and 37°C.

### Viability and metabolic activity

Viability was assessed with a live/dead Viability Kit (Live/Dead viability/cytotoxicity kit, Invitrogen) according to the manufacturer’s protocol. Beads were washed in phosphate buffered saline (PBS, Sigma) prior to staining. The cells in beads were then studied with Confocal Laser Scanning Microscope (LSM 510 META FCS, Zeiss) equipped with Argon (488nm) and a HeNe (543nm) lasers. The filters used were BP 505–530 and LP 650. Metabolic activity in the cells was evaluated by adding alamar blue assay (Invitrogen, Thermo Fischer Scientific, Waltham, MA, USA) by adding alamar blue reagent to a final concentration of 10% to beads corresponding to 5000 cells/well in a 96 well plate. The plate was incubated for 4 hours at room temperature before measuring fluorescence using a multilabel counter (Viktor 1420; Perkin Elmer, Wellesley, MA). The alamar blue reagent contains the weakly fluorescent indicator dye resazurin, which undergoes a colorimetric change, forming the highly fluorescent resorufin upon cellular metabolic reduction. Hence, the alamar blue assay is a non-toxic alternative to the commonly used MTT assay [21]. Moreover, the fluorescence readout is also not influenced by CaP minerals, which in our hands seemed to influence the absorbance readings in the MTT assay (not shown).

### Mineralization studies by LM and SEM

Mineralization of alginate beads was studied using light microscope (CKX41, Olympus). The mineral composite structure was also studied using Scanning Electron Microscopy (SEM). To prepare samples for SEM, beads with encapsulated cells were taken out of the culture flasks and mixed with Tissue Tek (Qiagen). Following, the beads were frozen using acetone precooled with liquid nitrogen in a small mould and thereafter cryo-microtomed (Leica CM 3050 S). To prepare the sections for imaging, the samples were dried in a critical point dryer (Emitech K850 Critical Point Dryer) and coated with 5nm Pt/Pd layer (80/20 using Cressington 208 HR sputter coater. Sections were examined using Hitachi S5500 scanning electron microscope operating at acceleration voltage of 5kV.

### RNA isolation, cDNA synthesis and RT-PCR

To isolate cells the beads were dissolved by using a *sequential* method described below as the conventional methods for cell recovery from alginate beads have been shown to affect cell viability [22]. Citrate (50mM, approximately 5mL) was added to the bead suspension and gently vortexed for about 5 minutes. At this point, the beads that were not yet dissolved were left to sediment to the bottom of the tube, and the rest of the suspension was pipette off and centrifuged (3min, 1000rpm). The remaining beads were given another 5mL of citrate and vortexed gently before a second round of sedimentation and centrifugation. This procedure was carried out until all beads were dissolved. Subsequently, recovered cells were washed with PBS, before a second round of centrifugation and supernatant removal. Relative gene expression quantification was carried out by Real-Time Quantitative PCR and TaqMan Gene Expression Reagents (Applied Biosystems Inc, Foster City, CA, USA) following the manufacturer's instructions. GAPDH was used as endogenous control. The samples were run in triplicates on StepOnePlus Real-Time PCR System (Applied Biosystems Inc, Foster City, CA, USA) and analyzed by the Applied Step One software 2.1. The following probes were used *RUNX2* (Hs00231692_m1, lot 959159), *SP7* (Hs00541729_m1, lot 961822), *BGLAP* (Hs01587814_g1, lot 969519) *COL1A1* (Hs 00164004_m1, lot 963537), *DMP-1*(Hs01009391_g1 lot 1048718), *SOST* (Hs00228830_m1, lot 997305) and (GAPDH, Hs99999905_m1, lot 853053). Genes with a Ct value >35 were considered as not detected.

### ALP assay

ALP activity was assesed using the ELF 97 Endogenous Phosphatase Detection Kit (Molecular Probes, Leiden, The Netherlands) according to the manufacturers protocol. At day 9 post encapsulation beads were dissolved in citrate (50mM) and cells were fixated and permeabilized before addition of the phosphatase substrate. Fluorescence was measured using a multilabel counter (Victor 1420, Perkin Elmer).

### Statistical analyses

To test for significant differences between groups two-way ANOVA followed by Sidak’s or Tukey’s multiple comparisons test was performed using GraphPad Prism version 6.00 for Mac OS X (GraphPad Software, La Jolla California USA).

## Results

### Scaffold characterization

**Fig 1:**
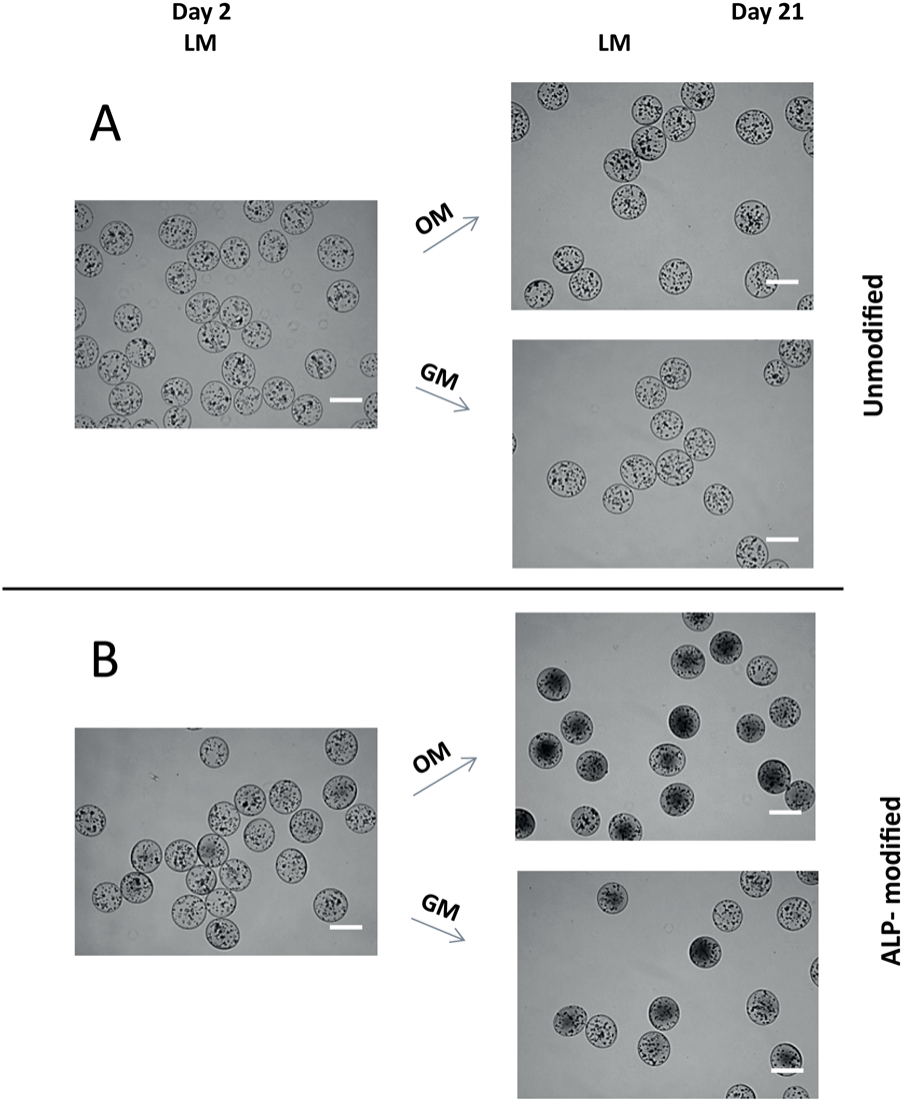
Mineralization of alginate beads. Light microscopy of unmodified (A) or ALP-modified (B) beads taken at day 2 and day 21 post encapsulation. Cells were cultured in either growth medium (GM) or osteogenic medium (OM). Mineralized beads appear dark whereas unmineralized beads appear transparent. Scale bar 500μm.

**Fig 2:**
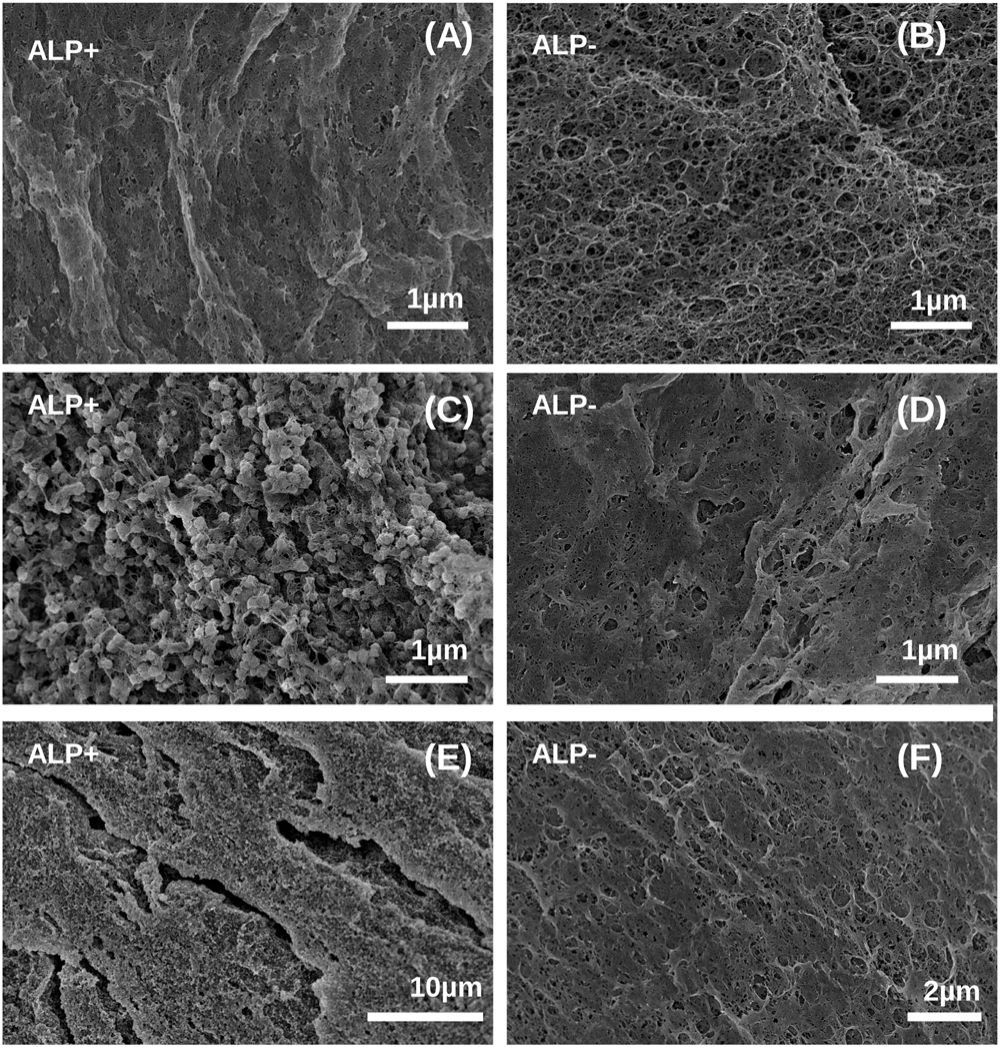
SEM micrographs of the alginate network in ALP-modified (A, C, E) or unmodified (B, D, F) beads taken at day 21 post encapsulation. Cells were cultured in either growth medium (A, B) or osteogenic medium (C-F). Mineral particles with spherical morphology are clearly visible for heavily mineralized samples shown at high and low magnification in panel C and E.

Mineralization of alginate beads can be observed by light microscopy as the beads become more opaque and appear dark under transmitted light [17]. Hence, we examined the beads with encapsulated MSC by light microscopy to evaluate mineralization at day 2 and 21 post encapsulation (Fig. 1). A total of 4 samples were studied: ALP-modified and unmodified beads cultured in either growth medium or osteogenic medium. At day 2 post encapsulation unmodified beads appeared relatively transparent (Fig. 1A). ALP-modified beads were mineralized as they appeared darker (Fig. 1B). Interestingly, beads which were initially ALP-modified were then further mineralized during culture in both osteogenic medium and growth medium (Fig. 1B). The unmodified beads, however, did only mineralize when they were cultured further in osteogenic medium, but to a lower extent than the ALP-modified beads. These findings suggest that MSC differentiate into osteogenic cells capable of inducing matrix mineralization in both modified and unmodified alginate beads. To examine the material in the beads in more detail, we examined beads that had been cultured for 21 days *in vitro* using scanning electron microscopy (SEM) (Fig. 2) We have previously shown that mineralization of alginate hydrogels can be characterized with SEM [17, 19]. In general, we observe mineral crystals in the samples that appeared mineralized by light microscopy. Hence, ALP-modified beads cultured in osteogenic medium had highest level of mineralization, showing distinctive granular crystal morphology (Fig. 2C and B). This is followed by the ALP-modified sample cultured in growth medium (Fig. 2A) and the unmodified beads cultured in osteogenic medium, where only some evidence of mineralization could be detected (Fig. 2D and F). In the unmodified sample cultured in growth medium no crystals could be seen either in LM or SEM. (Fig. 1A and 2B).

### Cell viability and metabolic activity

**Fig 3:**
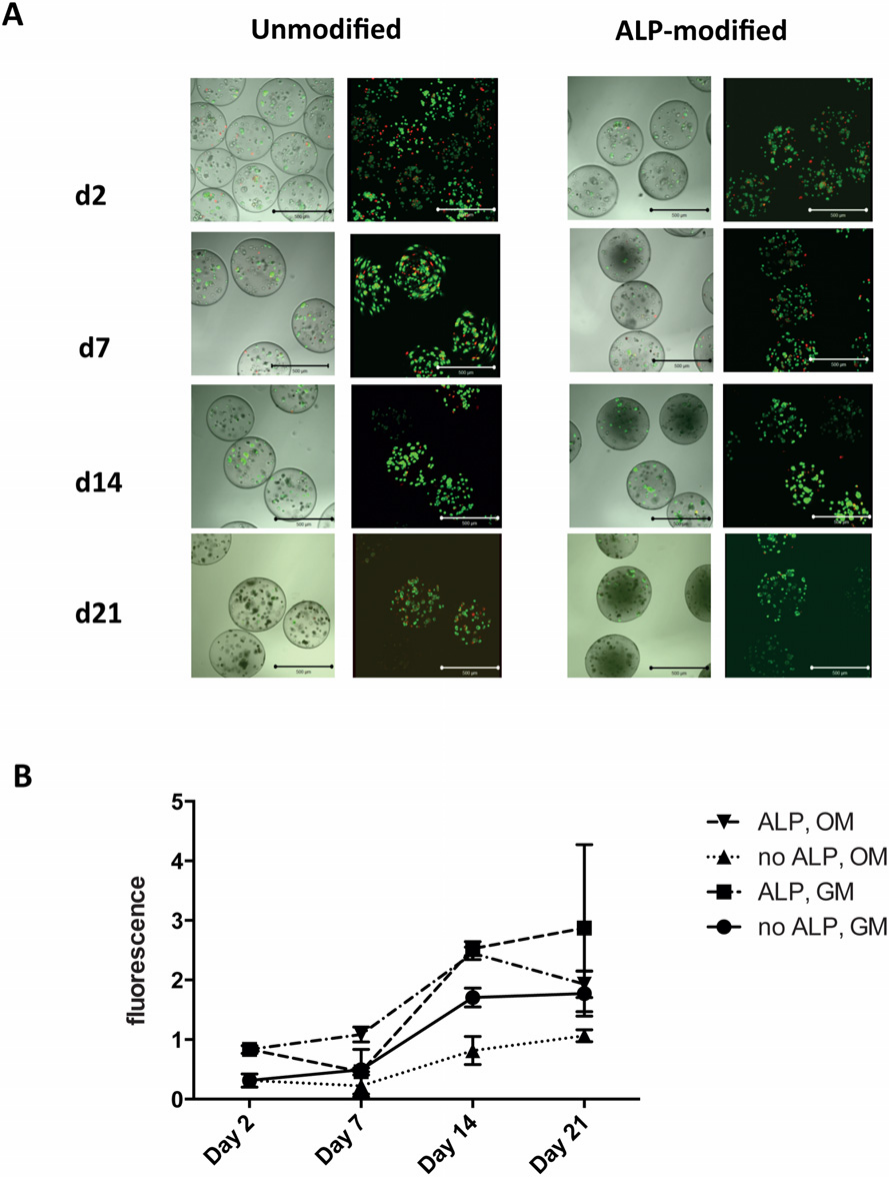
Viability of hMSCs in alginate beads cultured in osteogenic medium. **(A)** Live/dead fluorescent staining of cells cultured in osteogenic medium was visualized using confocal microscopy (LSM 510 META FCS, Zeiss). Left images: Confocal cross sections through overlaid transmitted light of hMSCs in alginate beads; Right images: three dimensional reconstructions of cross sections through the beads. Live cells appear green, dead cells appear red. Scale bar 500 μm. **(B)** Metabolic activity of MSC in alginate beads cultured in growth medium (GM) or osteogenic medium (OM) at day 2, 7, 14 and 21 post encapsulation measured by Alamar Blue assay. Metabolic activity was significantly different in ALP modified beads cultured in OM compared with unmodified beads cultured in OM at day 14, p <0.001, day 17, p< = 0.01 and day 21, p< = 0.001, Sidak’s multiple comparison test. The difference in metabolic activity between unmodified and ALP-modified beads cultured in GM was not statistically significant. The data presented are mean values +/- SD, n = 3. ALP: Containing 0.25mg/mL ALP.

Viability of the encapsulated cells was high after encapsulation and remained high with an estimated 90% viability for the duration of the study, as examined by visual inspection of live/dead staining by confocal microscopy (Fig. 3A). Moreover, no significant differences in viability were observed for the different sample groups (Fig. 3A and S1 Fig.). The metabolic activity measured by Alamar Blue assay increased over time for cells in both types of beads cultured in either OM and GM which further support that the cells survive in the beads, and might also suggest that the cells divide during the culture period (Fig. 3B). The metabolic activity appeared higher in cells cultured in growth medium compared with cells cultured in osteogenic medium, although the difference was only statistically significant at day 14 for cells cultured in unmodified beads (p≤ 0.05, Sidak’s multiple comparison test). The lower metabolic activity in cells cultured in OM might reflect a lower number of cells, as differentiated osteoblasts rarely divide compared with the more immature cells [23]. There was no significant difference in metabolic activity between cells cultured in GM in unmodified versus ALP-modified beads. However, at day 7, 14 and 21 after encapsulation, cells cultured in OM had a significantly higher metabolic activity in ALP-modified beads compared with cells in unmodified beads (p≤ 0.001, p≤ 0.01, p≤ 0.001, respectively, Sidak’s multiple comparison test). In conclusion, encapsulation of hMSC in mineralized and unmineralized alginate beads maintain high viability and metabolic activity.

### Collagen production

**Fig 4:**
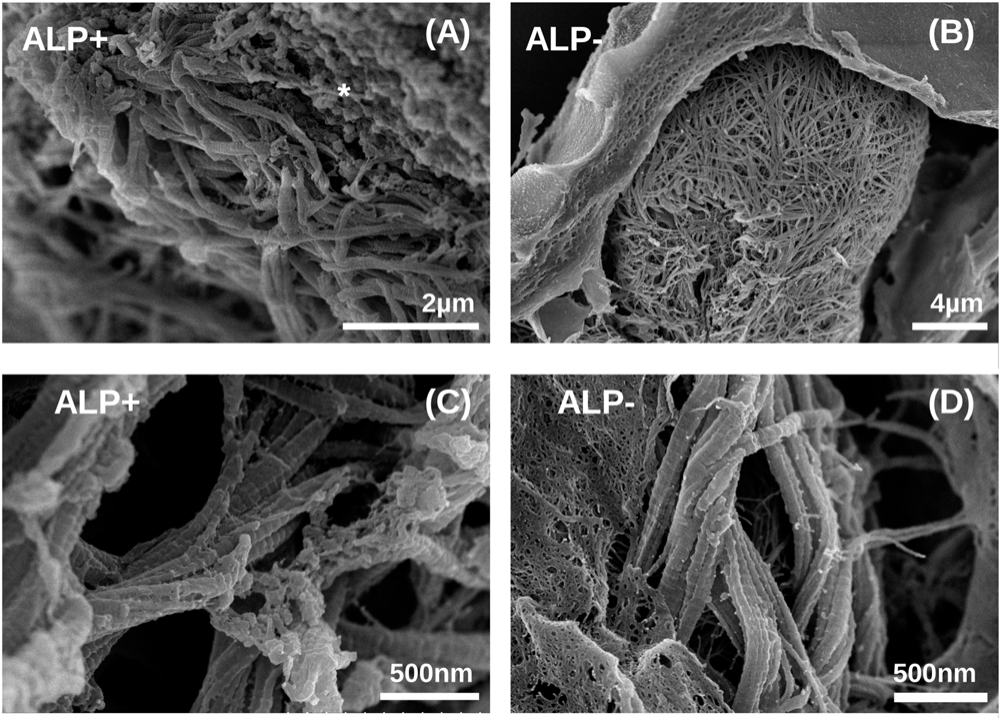
SEM micrographs of collagen fibrils in beads cultured in osteogenic medium. (A, C) low and high magnification of the space close to a cell producing collagen in ALP modified beads (ALP+). Mineral crystals similar to those shown in Fig. 2 are indicated by *; (B, D) low and high magnification micrographs of the space close to a cell producing collagen in unmineralized beads. Images were collected at 21 days post encapsulation.

To investigate whether the cells influenced the material properties during the culture period by e.g. secreting matrix proteins, beads from all 4 sample groups and cells from traditional culture plate (2D) samples were examined by SEM after 21days of culture. SEM microscopy revealed that cells in both ALP-modified (Fig. 4A and C) and unmodified alginate beads (Fig. 4B and D) cultured in osteogenic medium produce significant amounts of well organized, fibrillar collagen, which seemed to be located in the pericellular space between the cell surface and the alginate matrix (Fig. 4). The fibrils had diameters in the range of 100 nm and showed periodicity of 60–70 nm along the fibril axis typical for collagen type I (Fig. 4C and D). [24–26]. We did not observe collagen fibrils in beads cultured in growth medium (data not shown) suggesting that the 3D environment itself was not sufficient to promote collagen production. In the sample cultured in osteogenic medium on traditional culture plate (2D) no collagen fibrils was observed neither, but this could be due to it being washed away during media change or due to sample preparation for SEM (data not shown).

### Differentiation

**Fig 5:**
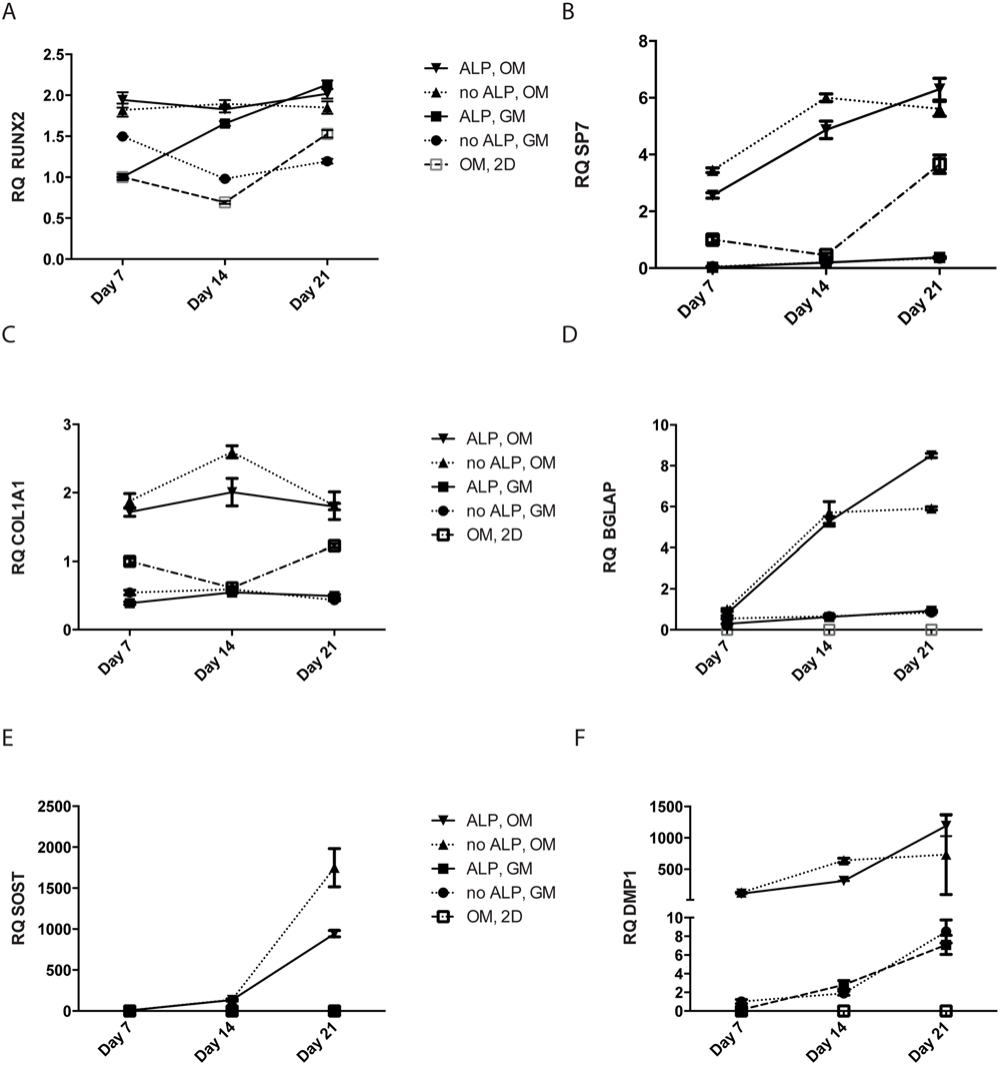
Relative mRNA expression of osteoblast/ osteocyte markers. MSCs were cultured in unmodified (no ALP), ALP-modified (ALP) alginate beads or on traditional culture plates (2D). Samples were cultured in either growth medium (GM) or osteogenic medium (OM) for 21 days post encapsulation. RUNX2 (A), Osterix (SP7) (B), COL1A1 (C), and sclerostin (SOST) (E) mRNA expression are relative to mRNA expression in cells cultured on traditional culture plates in osteogenic medium for 7 days. Osteocalcin (BGLAP) (D) and DMP1 (F) mRNA expressions are relative to mRNA expression in cells in unmodified alginate beads cultured in growth medium for 7 days post encapsulation. ND = not detected.

To characterize the differentiation stage of the encapsulated cells, mRNA expression of markers for osteoblast differentiation were examined at day 7, 14 and 21 post encapsulation. As a reference the gene expression levels in MSC cultured on tissue culture plates in osteogenic media for 7, 14 and 21 days were also quantified. *RUNX2* is considered an early osteoblastic gene [27], and we found *RUNX2* to be expressed in all samples (Fig. 5A). RUNX2 mRNA was significantly higher in ALP-modified beads cultured in GM compared with unmodified beads cultured in GM (p ≤ 0.001, all time points, Tukey’s multiple comparison test). However, there was no significant difference in RUNX2 expression in the different bead types cultured in OM. The expression of the transcription factor osterix (*SP7*) increases during osteoblast maturation [28]. We found that *SP7* was induced in cells cultured in traditional 2D in osteogenic medium; still, *SP7* expression was higher in cells cultured in OM in the two bead types compared with cells cultured in 2D (p ≤ 0.001, both bead types, all time points, Tukey’s multiple comparison test). In comparison, *SP7* expression in cells cultured in beads in GM was low, suggesting that a 3D environment by itself is not sufficient to promote the differentiation of fully mature osteoblasts (Fig. 5B). Collagen type I (*COL1A1*) is the major matrix protein produced by osteoblasts. Strikingly, cells cultured in osteogenic medium in both ALP-modified and non-modified alginate beads expressed higher levels of collagen type I than cells cultured in the same medium in traditional 2D, suggesting that a 3D environment might promote collagen type I expression (Tukey’s multiple comparison test p≤ 0.001 for both bead types) (Fig. 5C). At day 14, *COL1A1* was higher in unmodified beads compared with ALP-modified beads for cells cultured in OM (p ≤ 0.001, Tukey’s multiple comparison test) but in cells cultured in growth media COL1A1 mRNA expression was similar in the two bead types. Similarly, gene expression levels of osteocalcin (bone gamma-carboxyglutamic acid-containing protein (*BGLAP*)) a secreted protein produced by mature osteoblasts, were higher in cells cultured in osteogenic medium in 3D compared with cells cultured in osteogenic medium in traditional 2D culture, in which we could not detect *BGLAP* mRNA (Fig. 5D). There was no difference in *BGLAP* mRNA expression between cells cultured in GM in the two bead types; however, *BGLAP* was significantly higher in ALP-modified beads cultured for 21 days in OM (p ≤ 0.001, Tukey’s multiple comparison test).

**Fig 6:**
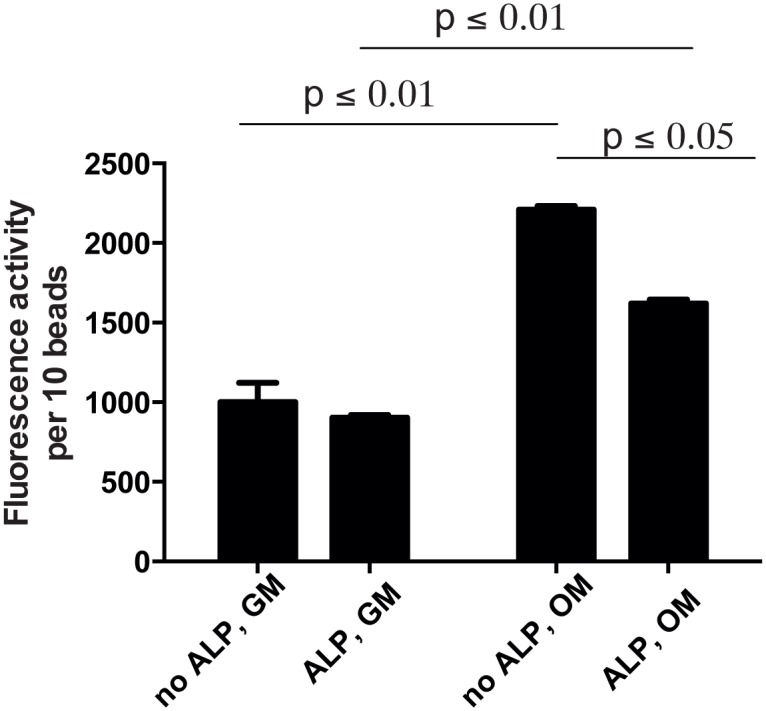
Alkaline phosphatase activity in cells cultured in ALP-modified or unmodified beads for 9 days as indicated. p values indicate statistical significant differences by Sidak’s multiple comparison test.

Sclerostin *(SOST)* is a marker specific for osteocytes/late osteoblasts and is induced after *BGLAP* [29]. Indeed, in cells cultured in OM in both bead types *SOST* mRNA was highly expressed (Fig. 4E), many hundred-fold increased compared with cells induced to differentiate on plastic. After 21 days, *SOST* mRNA was significantly higher in unmodified alginate beads compared with ALP-beads (p ≤ 0.001, Tukey’s multiple comparison test). Moreover, the mRNA expression of dentin matrix protein 1 (*DMP1*), an extracellular matrix protein expressed by osteocytes, was highly expressed in encapsulated cells cultured in OM, and was also expressed by cells in beads cultured in growth medium (Fig. 5F). After 21 days, *DMP1* was higher in ALP-modified beads compared with unmodified beads (p ≤ 0.05, Tukey’s multiple comparison test), whereas there was no difference in *DMP1* expression in cells cultured in GM. Cells cultured in osteogenic conditions on plastic did not express *DMP1*. To further verify that the cells differentiated in osteogenic direction, we measured alkaline phosphatase (ALP) activity by an enzymatic assay. Indeed, alkaline phosphatase activity increased in cells cultured in OM compared with GM in both ALP-modified and unmodified beads (p ≤0.01, Sidak’s multiple comparison test) (Fig. 6). ALP-activity also appeared higher in unmodified beads compared with modified beads cultured in OM (p ≤ 0.05, Sidak’s multiple comparison test), but not in GM.

Taken together, these results suggest that both unmodified and ALP-modified beads confer a 3D environment that promotes osteoblast differentiation and can support an osteocytic phenotype *in vitro*. The bead type, however, does not seem to have a major influence on osteogenic differentiation. Similar results were obtained for a second donor (S2 Fig.).

## Discussion

The main finding of this study is that enzymatic mineralization of alginate beads is compatible with the survival and osteogenic differentiation of encapsulated human MSC. Moreover, both ALP-modified and unmodified alginate beads promote osteogenic differentiation of MSC compared to differentiation in 2D. A greater extent of mineral deposition was found in the ALP-modified beads; hence these beads have the potential to serve as a bone substitute in tissue engineering.

Here, we show that human bone marrow-derived MSC cultured in both unmodified and ALP-modified alginate beads were efficiently induced to differentiate in osteogenic direction. These findings are in line with what has been shown for alginate beads in previous studies [30, 31]. However, the enzymatically mineralized alginate gels differs from already described models in that the matrix itself is bone-like as the mineral phase in previous studies has been characterized to be HA [19]. HA not only makes the material stronger and is more similar to bone, but it also may facilitate bone formation, both important features in bone regeneration scaffolds [32]. There are several alginate models where different types of calcium phosphates have been incorporated including HA [33] and calcium phosphate cement (CPC) [34–37]. These models all support growth and survival of pre-osteoblasts, but in contrast to our model the cells are (often) prepared separately from the minerals. Co-immobilizing cells with alkaline phosphatase (ALP) for gradual mineralization of the microenvironment is fundamentally different from seeding MSC into a macroporous scaffold and may provide a truer bone-like environment.

Another alginate model is the widely used RGD-model. RGD-modified alginate, in which the RGD peptides create attachment points for the encapsulated cells, has been shown to control phenotype, enhance cell-matrix interactions and promote differentiation into mature osteoblasts from mouse pre-osteoblasts [38], human pre-osteoblasts [39] and human MSC [30] The focus of these studies has been on cell characterization and although positive staining for calcium deposits has been shown in the RGD-alginate [30], no further material characterization has been proved. The enzymatic mineralization of the alginate gel allows for an even distribution of minerals throughout the matrix, where the deposition can be easily controlled by varying the concentrations of precursors and varying pH, temperature and buffer [19]. Mineralization of the ALP-modified beads continued for the duration of the study, regardless of culture medium. The mineral granules were in the 100 nm range and dispersed evenly in the alginate network. ALP activity assay performed at day 9 revealed that ALP was active only in cells cultured in osteogenic medium (data not shown). At day 21, unmodified beads cultured in osteogenic medium was mineralized, nevertheless to a much lower degree than ALP-modified samples regardless of culture medium. In the unmodified beads mineralization is a much slower process, only scarcely visible at day 21 post encapsulation. Hence, it seems that initial mineralization by the encapsulated ALP is important for further mineral deposition.

Interestingly, beads mineralized by encapsulated enzyme were quickly destabilized if the calcium concentration in the culture medium was too low. This could be stabilized by addition of 7.5 mM calcium to the culture medium. The mechanism of destabilization is likely due to the continuous activity of the enzyme liberating phosphate ions after the initial 48 hour mineralization period. As calcium is only present at low concentrations in the medium during the prolonged culture period, the calcium-alginate cross-links are destabilized and calcium ions bind, preferentially, to phosphate ions to form calcium phosphate. The proposed mechanism could provide a means of destabilizing the beads *in vivo* post implantation, which may be beneficial for the regeneration of bone at the implant site.

The encapsulated MSCs remained highly viable after encapsulation and for the duration of the study period (21 days). The high viability obtained in this study may be partially due to the small bead size making the nutrient, oxygen and waste distribution route short. This is an advantage as it will avoid the buildup of toxic waste products in the matrix. Additionally; the encapsulated cells remained metabolically active for the duration of the study. Although proliferation was not quantified in the present study no obvious increase in cell numbers was observed by visual inspection of encapsulated cells. However, an increase in metabolic activity in cells cultured in growth medium compared to the cells cultured in osteogenic medium probably reflects an increase in proliferation of undifferentiated osteoblasts compared with more mature cells [23]. Our results are in accordance with a study where MSC were seeded on top of BCP particles and cultured in either osteogenic or growth medium for 21 days. Results show that proliferation is lowest for cells cultured in osteogenic medium, regardless of modification, and highest when cultured in 2D [40].

Gene expression analyses indicated that the MSCs differentiated into mature osteoblasts and osteocytes. Early osteoblast markers were present in all samples, and the mRNA levels were highest in MSC cultured in beads in osteogenic medium, lower in MSC cultured on plates in osteogenic medium, and lowest in beads cultured in growth medium. Late osteoblast/osteocyte markers *(BGLAP, DMP1 and SOST)* were expressed only by cells in beads, which may infer that the 3D environment itself promote differentiation. Supporting this, we found that the osteocyte marker *DMP1* was only detectable in cells cultured in beads. Hence, the mineralized beads might resemble osteoid tissue where osteoblasts get buried into the tissue and become osteocytes [5]. Even though osteocytes are the most abundant cell in bone, they are, by far, the least studied. Until recently they were considered to be silent bystanders, but they have been shown to be important regulators of mineralization, sensing mechanical strain and maintaining bone homeostasis [41]. This highlights the importance of developing tools to study osteocytes *in vitro*. The differentiation towards osteocytes in bone tissue engineering systems is scarcely described. Our results suggest that mineralized alginate beads may be used as a model system.

In summary, our results demonstrate that enzymatically mineralized alginate beads are cell compatible, promote hMSC differentiation towards osteoblasts and further mineralization of the alginate matrix. Hence this is interesting for bone tissue engineering. Collagen type I fibrils were found inside beads cultured in osteogenic medium, and cells expressed both *BGLAP* and *DMP1*, which are both important for the mineralization of bone [42, 43]. Moreover, the alginate beads can also be used as tool to study osteoblastogenesis and osteocyte function *in vitro*.

## Supporting Information

S1 Fig Viability of MSCs in alginate beads cultured in growth medium. Live/dead stained cells were visualized using confocal microscopy (LSM 510 META FCS, Zeiss). Left images: Confocal cross sections through overlaid transmitted light of hMSCs in alginate beads; Right images: three dimensional reconstructions of cross sections through the beads. Live cells appear green, dead cells appear red. Scale bar 500μm.
(TIF)

S2 Fig Relative mRNA expression of osteoblast/ osteocyte markers. MSCs were cultured in unmodified (wo ALP), ALP-modified (ALP) alginate beads (3D) or on traditional culture plates (2D). Samples were cultured in either growth medium (GM) or differentiation medium (DM) for 21 days post encapsulation. mRNA expression of RUNX2 (A), COL1A1 (C) and osterix (B) are relative to cells cultured on traditional culture plates cultured in DM at d7 post encapsulation. DMP1 (D) mRNA expression is relative to mRNA expression in cells in unmodified alginate beads cultured in DM at d7 post encapsulation. ND = not detected.
(TIF)

S1 Table Immunophenotype of MSC purchased from Lonza Inc. (TIF)
