# Supplementary figures and images for: Osteogenic Differentiation of Human Mesenchymal Stem Cells in Mineralized Alginate Matrices
Source: PLoS One. 2015 Mar 13;10(3):e0120374. doi: 10.1371/journal.pone.0120374 (PMC4358956; doi:10.1371/journal.pone.0120374)

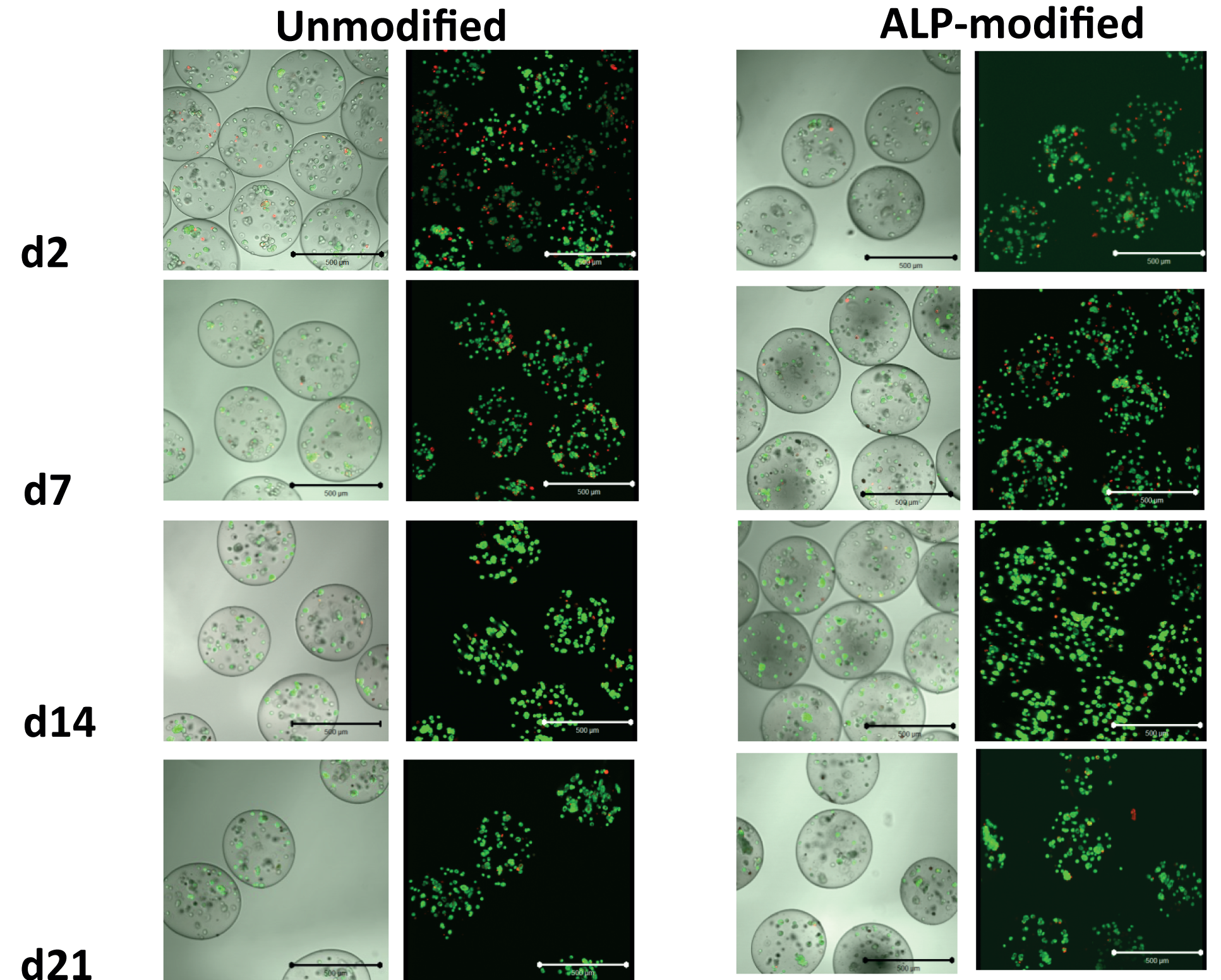

Supplement: S1 Fig — Live/dead stained cells were visualized using confocal microscopy (LSM 510 META FCS, Zeiss). Left images: Confocal cross sections through overlaid transmitted light of hMSCs in alginate beads; Right images: three dimensional reconstructions of cross sections through the beads. Live cells appear green, dead cells appear red. Scale bar 500μm. (TIF) [file pone.0120374.s001.tif]

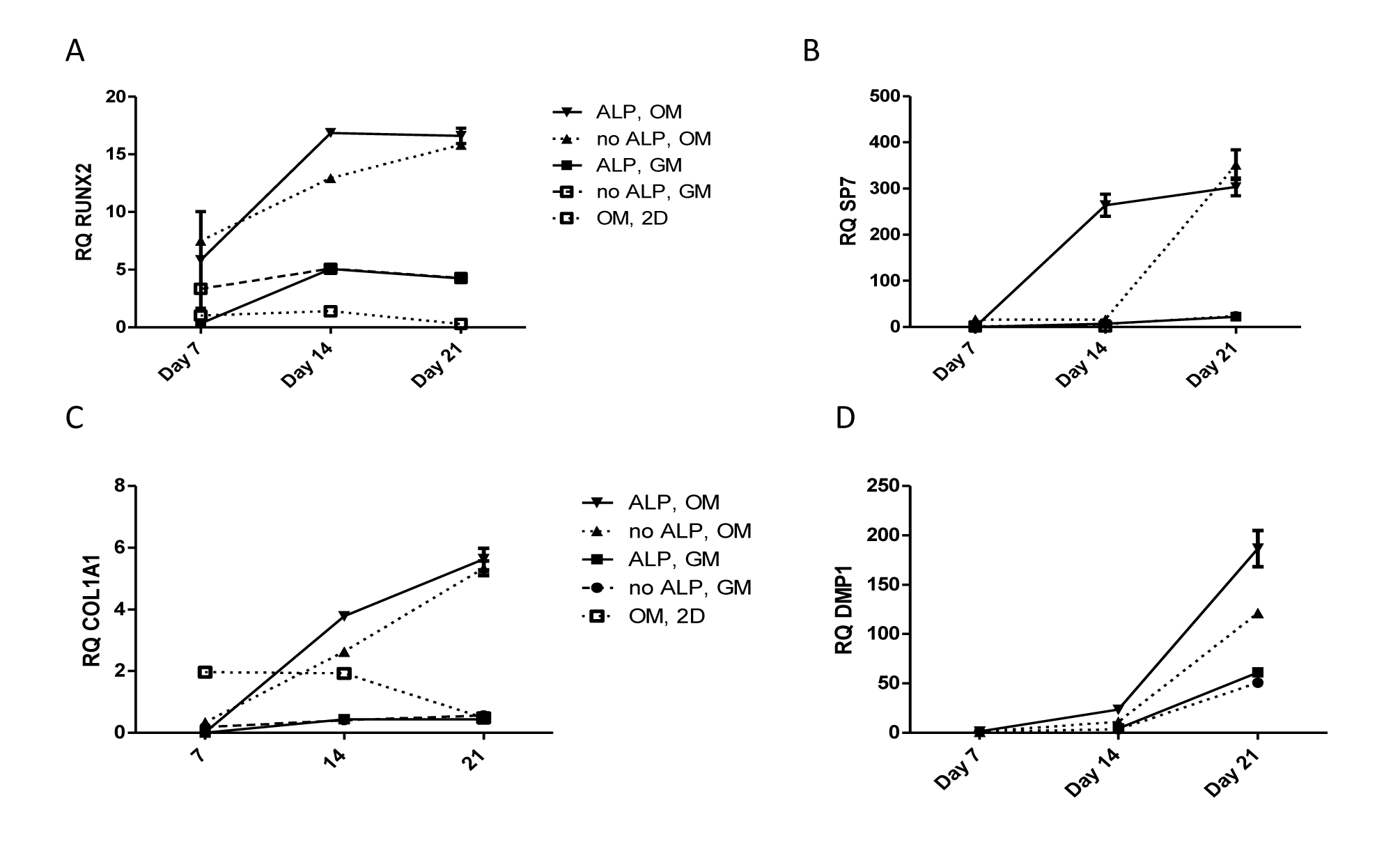

Supplement: S2 Fig — MSCs were cultured in unmodified (wo ALP), ALP-modified (ALP) alginate beads (3D) or on traditional culture plates (2D). Samples were cultured in either growth medium (GM) or differentiation medium (DM) for 21 days post encapsulation. mRNA expression of RUNX2 (A), COL1A1 (C) and osterix (B) are relative to cells cultured on traditional culture plates cultured in DM at d7 post encapsulation. DMP1 (D) mRNA expression is relative to mRNA expression in cells in unmodified alginate beads cultured in DM at d7 post encapsulation. ND = not detected. (TIF) [file pone.0120374.s002.tif]

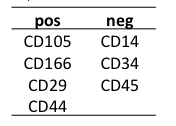

Supplement: S1 Table — (TIF) [file pone.0120374.s003.tif]
